# Antagonist Anti-CD28 Therapeutics for the Treatment of Autoimmune Disorders

**DOI:** 10.3390/antib6040019

**Published:** 2017-11-21

**Authors:** Bernard Vanhove, Nicolas Poirier, Fadi Fakhouri, Laetitia Laurent, Bert ’t Hart, Pedro H. Papotto, Luiz V. Rizzo, Masaaki Zaitsu, Fadi Issa, Kathryn Wood, Jean-Paul Soulillou, Gilles Blancho

**Affiliations:** 1 OSE Immunotherapeutics, 44200 Nantes, France; nicolas.poirier@ose-immuno.com; 2 Centre de Recherche en Transplantation et Immunologie (CRTI) UMR1064, INSERM, Université de Nantes, 44035 Nantes, France; Fadi.FAKHOURI@chu-nantes.fr (F.F.); laetitialaur@gmail.com (L.L.); soulillou@yahoo.fr (J.-P.S.); gilles.blancho@chu-nantes.fr (G.B.); 3 Institut de Transplantation Urologie Néphrologie (ITUN), CHU Nantes, 44093 Nantes, France; 4 Biomedical Primate Research Centre, 2288 GJ Rijswijk, The Netherlands; hart@bprc.nl; 5 Department Neuroscience, University of Groningen, University Medical Center, 9713 GZ Groningen, The Netherlands; 6 Instituto de Medicina Molecular, Faculdade de Medicina, Universidade de Lisboa, 1649-004 Lisbon, Portugal; pedro.papotto@gmail.com; 7 Hospital Israelita Albert Einstein, Av. Albert Einstein 627-701, 2-SS Bloco A, 05651-901 São Paulo, Brazil; lvrizzo@einstein.br; 8 Nuffield Department of Surgical Sciences, University of Oxford, Oxford OX3 9DU, UK; m.zaitus@gmail.com (M.Z.); fadi.issa@nds.ox.ac.uk (F.I.); kathryn.wood@nds.ox.ac.uk (K.W.)

**Keywords:** autoimmunity, T cell costimulation, antibodies

## Abstract

The effector functions of T lymphocytes are responsible for most autoimmune disorders and act by directly damaging tissues or by indirectly promoting inflammation and antibody responses. Co-stimulatory and co-inhibitory T cell receptor molecules are the primary pharmacological targets that enable interference with immune-mediated diseases. Among these, selective CD28 antagonists have drawn special interest, since they tip the co-stimulation/co-inhibition balance towards efficiently inhibiting effector T cells while promoting suppression by pre-existing regulatory T-cells. After having demonstrated outstanding therapeutic efficacy in multiple models of autoimmunity, inflammation and transplantation, and safety in phase-I studies in humans, selective CD28 antagonists are currently in early clinical development for the treatment of systemic lupus erythematous and rheumatoid arthritis. Here, we review the available proof of concept studies for CD28 antagonists in autoimmunity, with a special focus on the mechanisms of action.

## 1. Introduction

Autoimmunity arises when self-antigens or modified self-antigens are presented to T lymphocytes in the absence of appropriate retro control. T cells become activated after integration of three types of signals. T cell receptor (TCR) signaling triggered by a peptide antigen/HLA complex presented by an antigen-presenting cell (signal 1). This is either reinforced or dampened by engagement of co-stimulatory/co-inhibitory molecules (signal 2) and cytokines (signal 3) that regulate T cell differentiation into pathogenic effector T cells (Teff), anti-inflammatory regulatory T cells (Treg), or memory T cells. 

**Figure 1:**
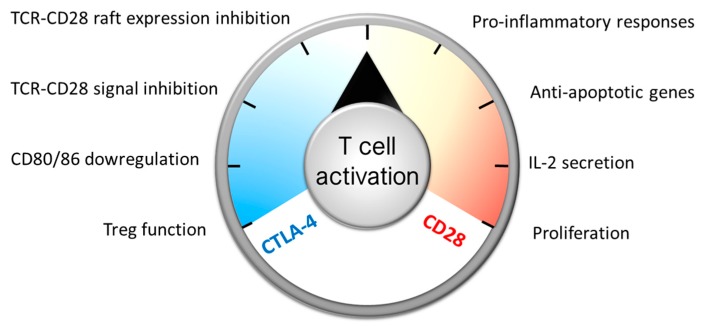
While many T cell co-stimulatory systems have been described, the main molecules driving de novo T cell responses are CD28 and CTLA-4. CD28 binds to CD80/86 expressed on antigen-presenting cells and “warms up” T cells (through the activation of pro-inflammatory, pro-survival, and activating and proliferation factors), whereas CTLA-4 “cools it down” by counteracting CD28-mediated events, by eliminating CD80 molecules on antigen-presenting cells and by activating negative regulators.

Whereas many T cell costimulatory/co-inhibitory systems have been identified, one of the most important checkpoints controlling initial T cell differentiation is the interaction of CD80/86 on antigen presenting cells (APC), with CD28 and CTLA-4 on T cells acting as a rheostat to turn T cells on and off (Figure 1). Signal 1 plus CD28-mediated co-stimulation results in T cell activation, proliferation, the synthesis of anti-apoptotic genes, and pro-inflammatory responses, and enhances IL-2 mRNA transcript stability and IL-2 secretion, which is necessary for effector T cell and Treg expansion [1]. CTLA-4 is upregulated on naïve T cells shortly after activation or constitutively expressed on Treg cells. It prevents CD28-mediated signals by two major mechanisms: [1] it can act by down-regulating CD80 and CD86 on antigen presenting cells (APCs) by trans-endocytosis, thereby altering the level of CD28 engagement [2]. In particular, follicular T helper cell (Tfh) differentiation is regulated by graded control of CTLA-4 by the strength of CD28 engagement [3]. (2) CTLA-4 has also been described to act directly in a cell-intrinsic manner by recruiting phosphatases opposing TCR and CD28-mediated signals [4], and by inhibiting TCR–CD28-mediated raft expression [5]. The clearest proof indicating that CTLA-4 is a major checkpoint for T cells comes from the observation that knocking out *ctla4* in mice leads to a lethal lymphoproliferative syndrome [6,7], whilst in humans some immune dysregulation (such as Graves’ disease, autoimmune hypothyroidism, and type 1 diabetes) [8] and lymphoproliferative diseases [9] result from genetic alterations that cause CTLA-4 deficiency. 

The first biologics used to interfere with CD28-mediated signals were wild-type or high-affinity, recombinant-soluble domains of CTLA-4 fused with an immunoglobulin Fc domain (e.g., many forms of labscale CTLA4-Ig, abatacept, and belatacept). CTLA4-Ig molecules dock onto CD80 and CD86 and therefore inhibit binding of CD28. By doing so, CTLA4-Ig molecules have the capacity to also inhibit binding of CTLA-4 to CD80/86 (and also of CD80 to PD-L1 [10]), which might perturb the co-inhibitory function of membrane-bound CTLA-4. Indeed, a reduced accessibility of CTLA-4 for CD80/86, with CTLA4-Ig or other reagents, led experimentally in vitro [11,12] and in vivo [13,14,15] to a reduction of the suppressive functions of Treg cells. Therefore, we and others proposed [16,17] that selectively targeting CD28 might share the benefit of CTLA4-Ig (blockade of CD28-mediated signals) without perturbing the co-inhibitory CD80/86-CTLA4 axis required for the control of Treg cell functions and for the control of effector T cells, particularly for highly differentiated effector memory T cells (such as Th17 cells), which are tightly controlled by CTLA-4 [18]. Since then, the “selective CD28 blockade” proof of concept has been tested in experimental transplantation and autoimmune settings and has also begun evaluation in humans. 

Blocking the CD28-CD80/CD86 pathway using anti-CD28 monoclonal antibodies (mAbs) has been challenging due to the inadvertent stimulatory activity of most conventional anti-CD28 mAbs (for review [19]). More precisely, because CD28 is expressed on cell membranes as homodimers, anti-CD28 antibodies, which are also homodimeric molecules, induce a clustering of CD28 molecules, which results in the phosphorylation of PI3K, a molecular signal also induced by engagement of CD80/86 [20]. This occurs independently of the binding epitope, so that a given anti-CD28 antibody can be an antagonist of CD80/86 (if it binds to the MYPPPY domain recognized by CD80/86) while still presenting agonistic properties. To date, all antibodies directed against CD28 were found to activate the receptor instead of only blocking access to its ligand. An exception is the anti-rat JJ319 mAb, which in vivo rapidly induces internalization of CD28 and presents functional antagonist properties [21]. To our knowledge, an antibody-inducing CD28 downmodulation has not been identified in another species. “Superagonistic” anti-CD28 antibodies (such as the TGN1412 antibody [22]) bind to the laterally exposed C’’D loop of CD28 and induce a non-physiological engagement of CD28 resulting in polyclonal T cell activation and cytokine release even in the absence of TCR stimulation [23]. To develop “antagonist-only” anti-CD28 antibodies, mutations in the Fc domain have been introduced to prevent cross-linking of CD28 through Fc/FcγR interaction. However, while this strategy was efficient in vivo in rodents [24,25], “Fc-silenced” anti-CD28 mAb still co-stimulated human T cell proliferation and cytokine release in vitro [26], which halted their clinical development.

In our first studies specifically targeting CD28, we also introduced a strategy that aimed at avoiding crosslinking CD28 and described a monovalent anti-CD28 antibody fragment (i.e., Fab or scFv), presenting an “antagonist-only” action on T cells [16,20]. To increase the otherwise limited in vivo half-life of monovalent antibody fragments, molecular fusions with alpha-1-antitrypsin [16] or chemical conjugation with a polyethylene glycol (PEG) moiety have been proposed [17,27]. While single-chain-Fv-alpha-1-antitrypsin conjugates still presented a limited half-life in primates of about 24 h [11], conjugation of a Fab’ antibody fragment with PEG resulted in a weeklong, clinically compatible extension in primates and in humans [17,27,28]. These pegylated monovalent anti-CD28 antibodies presented a remarkable safety profile in pre-clinical models [19] and in humans [28]. To our knowledge, two “antagonist-only” anti-CD28 Fab’-PEG antibodies are currently in clinical development: FR104 (OSE Immunotherapeutics, Nantes, France/ Janssen Inc. Springfield, PA, USA) and the lulizumab (BMS-931699; Bristol Myers Squibb, New-York City, NY, USA).

Here, we review available preclinical experience with “antagonist only” anti-CD28 antibodies in autoimmunity models and discuss what clinical benefit might be obtained from this novel therapeutic approach in the corresponding human pathologies. 

## 2. Models of Immune-Mediated Diseases of the Skin

The skin is a large and complex organ whose function by far exceeds a barrier against external insults. The skin also tightly regulates inflammatory responses and supports immunity against infections, allergens, and tumors. Diverse innate and adaptive inflammatory and regulatory immune cells collaborate to maintain immune skin homeostasis [29]. Inflammatory skin disorders are quite diverse, and result in a wide range of symptoms ranging in severity from mild itching and redness to serious medical health complications in chronic conditions such as psoriasis. Psoriasis is a complex multifactorial relapsing inflammatory skin disease characterized by erythematous scaly plaques [30]. Psoriasis lesions are characterized by histological features such as a thickened epidermis caused by keratinocyte proliferation (acanthosis), the retention of nuclei in the stratum corneum (parakeratosis) that arises from an aberrant differentiation of keratinocytes, and inflammatory cell infiltrates in the epidermis and dermis. Despite intensive research and the identification of the significant role of Th17 cells, the underlying pathogenic mechanisms remain to be fully understood. Whilst the pharmacological treatment of psoriasis has substantially progressed over the last decade, novel drugs currently do not satisfy long-term efficacy and safety requirements [31,32]. Blocking costimulation at the origin of the expansion/activation of pathogenic effector T cells while preserving natural co-inhibition and regulatory T cell functions might result in long-term remission.

CTLA4-Ig (Abatacept, Bristol Myers Squibb, New-York City, NY, USA) has been previously evaluated in a phase 1 open-label dose-escalation study in psoriasis vulgaris [33]. Forty-six percent of all study patients achieved a 50% or greater sustained improvement in clinical disease activity, with progressively greater effects observed in the highest-dosed cohorts. Recently, Abatacept demonstrated efficacy in a double-blind placebo-controlled phase III trial in psoriatic arthritis patients but with only modest benefit on psoriatic skin lesions [34]. Due to the mixed mechanism of action of CTLA4-Ig on both costimulatory and co-inhibitory pathways, as well as the greater sensitivity of Th17 to co-inhibition by CTLA-4 [35], several groups evaluated the efficacy of selective CD28 antagonists in the context of skin inflammation (DTH, Transplantation) and, more particularly, psoriasis.

Humanized mouse skin transplantation models provide a useful method for directly assessing human skin as a T cell target. FK734, an Fc-silenced anti-CD28 mAb, demonstrated preclinical efficacy in a humanized model in which human psoriasis plaque from patients were grafted onto immunodeficient SCID mice [25]. FK734 could reduce the epidermal thickness and the magnitude of human lymphocytic infiltrates in these transplanted human psoriasis plaques. It was also efficient at preventing human skin (from healthy volunteers) allograft rejection in humanized mice, in which it reduced endothelial injury and thrombosis, as well as human T cell skin infiltration [26]. However, as previously described, this FK734 antibody shared both antagonist and agonist properties, and its clinical development was stopped. While models of skin grafts on humanized mice are usually alloimmune rather than autoimmune, they are particularly stringent and offer ‘human-specific’ mechanistic insights that cannot be gained elsewhere. In the context of transplantation, it is important to note that CTLA4-Ig has not gained a large amount of traction due to the comparatively high acute rejection rates in renal transplantation [36,37]. In a recent experimental study examining alloimmune responses against human skin, FR104 was compared directly to CTLA4-Ig [15]. Of interest, both agents blocked T cell proliferation and activation in vitro, yet in vivo in a humanized model only FR104 demonstrated potent suppression of T cell responses against human skin. This was associated with a reduction in the expression of the cutaneous lymphocyte antigen (CLA) molecule on circulating T cells, thus reducing their access to skin. Infiltration of human skin grafts with CD8+ effector T cells has previously been shown to be important for the destructive alloimmune response in this model [38]. Much of the enhanced effectiveness of FR104 over CTLA4-Ig may be attributed to its ability to maintain immune regulation, as evidenced by the preservation of FOXP3+ cell infiltration into skin, both in this model and others [39]. Indeed, CLA+ regulatory T cell (Treg) infiltration into skin has previously been shown to be beneficial for the protection of skin against T cell responses [40]. An intriguing aspect of these findings in this and other humanized models is that direct CD28 blockade, but not CTLA4-Ig treatment, is able to terminate immune responses that are known to be mediated by memory phenotype T cells [41]. This may be because depriving the evolving alloimmune response of CTLA-4-mediated co-inhibition results in an inability to terminate T cell activation [42]. In both autoimmune and alloimmune pathology, the skew towards effector activity is associated with reduced immune regulatory activity. Maintaining the ability of Tregs to exert activity through one of their principal effector molecules, CTLA-4, is therefore important [43,44]. In support of this is the finding that human skin transplants receive enhanced protection from alloimmune destruction when adoptive Treg therapy is combined with FR104, but not CTLA4-Ig. In vitro, this effect manifests as effector T cell resistance to Treg-mediated suppression when under the direct influence of CTLA4-Ig, but not FR104. The ability to maintain suppression in the presence of direct CD28 blockade highlights the role that Treg-expressed CTLA-4 plays in promoting co-inhibition through parallel mechanisms. 

Besides using human skin grafts on humanized mice as a surrogate model for skin inflammatory disorders, novel experimental models of skin inflammation have been recently developed based on clinical evidence in humans. Aldara cream, containing 5% Imiquimod (a TLR7 and TLR8 agonist), is used in humans for topical treatment of genital and perianal warts, actinic keratosis, and superficial basal cell carcinoma. Several studies have, however, reported that Imiquimod application induces psoriasis or exacerbates the disease in patients, and that psoriasis-like lesions occur at both the treated area but also at distant skin sites that were unaffected before treatment [45,46,47,48]. Topical treatment of mouse skin with Aldara was reported as a novel mouse skin inflammatory model with histology that closely resembles psoriasis inducing acanthosis, parakeratosis, and a mixed inflammatory infiltrate with a predominance of the IL23/IL-17 axis similar to humans [49,50,51]. We found that daily topical application over 2 weeks to baboons also induced chronic erythema, as well as skin thickening and scaling, with a histology which resembled psoriasis and lichenoid lesions, and with a predominance of the IL-23/IL-17 molecular axis [52]. Skin inflammation resolved spontaneously after arrest of Aldara application, allowing us to perform different cycles of Aldara over months to model relapse phases in patients. A single administration of FR104 at 10 mg/kg was performed on day 0 of Aldara application. FR104 significantly reduced skin erythema, skin thickening, and desquamation. Epidermal thickening and proliferation, as well as T-cell and macrophage skin infiltrates, were also strongly decreased in line with clinical improvement. The protective clinical and biological benefit of FR104 was still observable during the second cycle of Aldara application performed two months after FR104 injection, demonstrating that FR104 has a long-term action after a single administration. 

To address the impact of selective CD28 blockade more specifically on memory T lymphocytes, we used more conventional delayed-type hypersensitivity (DTH) models of skin inflammation. DTH is clearly different from psoriasis as it is characterized as a type IV hypersensitivity reaction, but involves cell-mediated immunity initiated by CD4 and CD8 antigen-specific memory Th1 lymphocytes secreting IFNγ and the recruitment of macrophages [53]. Before the discovery of the importance of the IL-17 axis in psoriasis, Th1 responses were considered as a hallmark of psoriasis in opposition to the Th2-bias in atopic dermatitis. We previously developed a DTH model in baboons immunized twice with Bacillus Calmette–Guérin (BCG) vaccine and then chronically challenged by intradermal injection of tuberculin to induce erythema and skin inflammation characterized by infiltration by macrophages and memory T lymphocytes [54]. Like the Aldara model, skin inflammation resolved spontaneously, which allowed us to perform new skin antigen re-challenge every month to mimic relapse phases in patients. FR104 dose-dependently suppressed memory T cell-induced skin inflammation [55], with a very long-term antigen-specific hyporesponsiveness effect at 10 mg/Kg, even several months after complete drug elimination. Interestingly, CTLA4-Ig (Belatacept, Bristol Myers Squibb, New-York City, NY, USA) had no effect on this memory-driven model, once again demonstrating the advantage of selectively targeting CD28 while sparing co-inhibition for long-term effect. Importantly, we found that FR104 does not impair memory immunity against chronic latent virus infection, since no reactivation against several viruses relevant to human immune-related complications have been observed in treated animals, such as Hepatitis E virus (HEV), polyomavirus (SA12, SV40), Herpes virus (HVP-2), Cytomegalovirus (CMV), and Lymphocryptovirus (LCV, closely related to human EBV).

## 3. Models for Neuroinflammatory Diseases

Experimental autoimmune encephalomyelitis (EAE) is an accepted model of the human autoimmune component of the neurological disease multiple sclerosis (MS). EAE models in genetically susceptible strains of inbred/SPF mice and rats and non-human primates are used for translational research into the pathogenesis and therapy of MS [56]. New World primates, such as the common marmoset (*Callithrix jacchus*), and Old World primates, such as the rhesus monkey (*Macaca mulatta*) and the cynomolgus monkey (*Macaca fascicularis*), appear to be equally susceptible to EAE, but differ dramatically in clinical and pathological presentation. While the EAE model in marmosets approximates chronic MS in clinical and pathological presentation, the EAE models in both macaque species more closely resemble acute post-infectious forms of human demyelinating disease, such as acute disseminating encephalomyelitis. Because FR104 lacks cross-reactivity with marmoset CD28, Haanstra et al. tested the EAE model in rhesus monkeys rather than testing in marmoset models [57]. EAE was induced in 12 healthy adult male rhesus monkeys by a single intracutaneous immunization with recombinant human myelin oligodendrocyte glycoprotein (residues 1-125; rhMOG) formulated with CFA (day 0). This model has been validated with the anti-α4β1 mAb Natalizumab, which is clinically used for the treatment of MS [58]. The 12 monkeys were randomly assigned to the treatment of control group (both *n* = 6). FR104, or the equivalent volume of the solvent vehicle, were dosed via the intravenous route at 10 mg/kg on days 0, 7, 14, and 21. Pharmacokinetic assessment showed that adequate trough levels (>50 µg/mL) of FR104 were achieved throughout the study and that FR104 was remarkably non-immunogenic despite the usage of the strong bacterial adjuvant CFA. The clinical effect was 100% as 6/6 placebo-treated monkeys succumbed to severe clinical EAE, whereas 0/6 FR104-treated monkeys developed clinical symptoms. The dramatic clinical effect was reflected at the pathological level, whereby cerebral inflammation and demyelination were strongly suppressed. A potential adverse effect of strong immune suppression is the reactivation of latent viral infections such polyoma- and herpes-viruses, which these monkeys are naturally infected with. Overall, no significant reactivation of CMV or polyomavirus (SV40, SA12) was observed, but elevated levels of the EBV-related Macaca lymphocryptovirus were detected in the FR104-treated monkeys [57]. 

An often-heard criticism against the preclinical relevance of animal disease models is that the experimental treatment acts on the activation of naïve T cells, which is fundamentally different from the situation in patients where the autoreactive T cells are antigen-experienced and often constitutively activated. Recent work in the marmoset EAE model shows that the disease is not driven by immunologically naïve T cells, but by antigen-experienced effector memory T cells present in the pathogen-educated primate immune system. Although this observation needs to be replicated for the rhesus monkey, it suggests that the primate model more closely resembles the clinical situation. 

## 4. Arthritis Models

Type II collagen-induced arthritis (CIA) is an accepted model of human arthritic disease. CIA models have been established in genetically susceptible strains of inbred/SPF mice and in marmosets, rhesus monkeys, and cynomolgus monkeys. In the past two decades, the rhesus monkey model of CIA has been used for a variety of purposes, including the analysis of critical autoimmune mechanisms, the identification of biomarkers for the two main aspects of the disease, namely the inflammation and erosion of the synovial joints, and, finally, the efficacy assessment of a broad range of promising new treatments. These include small molecules, cytokines, mAbs, and gene therapy. In a blinded and placebo-controlled preclinical study, Vierboom et al. compared FR104 versus CTLA4-Ig (Abatacept) efficacy in a collagen-induced arthritis (CIA) monkey model [59]. CIA was induced by intracutaneous immunization with chicken collagen type II (chCII) in complete Freund’s adjuvant. FR104 and Abatacept were administrated at 10 mg/kg on day 0, 7, 14, 21, 28, 35, and 42. Both drugs significantly suppressed clinical symptoms of arthritis, C-reactive protein (CRP) and IL-6 inflammatory biomarkers, and anti-collagen type II serum antibody responses (both IgM and IgG). Joint inflammation, as well as bone and cartilage damage, were also significantly suppressed with both FR104 and Abatacept. In contrast to Abatacept, however, only FR104 showed effective suppression of chCII-induced peripheral blood monocytic cells proliferation, in particular in lymph nodes. It is possible that the difference between Abatacept and FR104 lies in the better control by FR104 of follicular T helper cells (Tfh) in the lymph nodes. Indeed, Tfh express high levels of PD-1 and ICOS, which, in addition to their respective ligands PD-L1 and ICOS-L, also interact with CD80 and CD28, respectively [10,60]. On one hand, Abatacept limits the co-inhibitory interaction between PD-1 and CD80, and, on the other hand, FR104 blunts the co-stimulatory interactions between ICOS and CD28 [27]. Ville et al. have shown in both mice and primates that Tfh responses and the release of IL-21, in a situation where CD28-CD80/86 interactions are similarly inhibited, are more repressed when PD-1/CD80 interactions are unopposed and CD28/ICOS interactions are blocked [61].

## 5. Autoimmune Uveitis Model

Autoimmune uveitis (AIU) is part of a wide range of diseases characterized by inflammatory responses in the uvea, an anatomical layer of the eye located between the sclera and the retina including the iris, ciliary body, and choroid. Inflammation can also extend to adjacent tissues, such as the retina, optic nerve, and vitreous humor [62]. AIU affects people in their most productive years from 20 to 50 years of age. It is among the leading causes of visual deficit and blindness [63] (approximately 10% of all cases), and has a very significant impact on quality of life of these patients. Moreover, some autoimmune diseases such as Behçet’s disease [64], reactive arthritis [65], sarcoidosis [66], and Vogt-Koyanagi-Harada syndrome [67] may additionally exhibit uveitis along with other clinical manifestations; however, eye disease exhibits particularities in these syndromes. 

Current therapies for AIU rely mainly on immunosuppression strategies to control acute inflammation and to ensure the maintenance of long-term remission. Corticosteroids are usually the first line of treatment due to their effectiveness at controlling inflammation, both in the short term and in the long term. However, a myriad of possible side effects (e.g., weight gain, gastric ulceration, osteoporosis, fluid retention, hypertension, diabetes mellitus, and changes in mental status), as well as ocular sequelae (e.g., acceleration of cataract formation and glaucoma), may be observed [68]. Although a number of promising strategies are being developed, most have limited efficacy, given the heterogeneous nature of AIU. Blockade of IL-2R with Daclizumab, for example, is not effective in all cases of anterior uveitis [69]. Furthermore, whilst TNF-α antagonists are well tolerated, they are only efficient in some specific cases of uveitis [70]. Altogether, the difficulties of targeting cytokines in AIU highlight the non-redundant roles of different cytokines in the pathogenesis of the disease. 

A significant effort has been undertaken to discover new targets and molecules that would allow for more precise manipulation of the immune system with the additional goal of mitigating side effects. In this regard, the use of different animal models for uveitis [71] has been key in the development of novel therapeutic approaches. Among the many animal models available, experimental autoimmune uveitis (EAU) is the most accepted model for human AIU, since EAU shares key characteristics with its human counterpart, such as the nature of antigens, T cell involvement, and histological features. The disease model is initiated by immunization of rodents with ocular antigens—such as the interphotoreceptor retinoid-binding protein (IRBP) or its immune-dominant epitopes—in the presence of adjuvants [72]. This process leads to the onset of T-cell-mediated ocular inflammation, with cellular features resembling those of human AIU. Infiltrating CD4^+^ T cells express a mainly T helper (T_H_) 1 phenotype [73], but Th17 cells were also shown to play an important role in the EAU pathogenesis [74]. Importantly, both T_H_ subsets are able to independently promote the onset of the disease [75].

Using EAU to mimic human AIU, Papotto and colleagues showed that administration of mPEG PV1 Fab’ (referred to as PV1 hereafter) during the initial phases of disease decreased ocular tissue damage and disease incidence [76], corroborating previous findings that pointed to B7/CD28 blockade as a promising immunotherapeutic strategy for EAU [77]. The observed reduction in ocular inflammation was accompanied by a decrease in the activation profile of CD4^+^ and CD8^+^ eye-infiltrating T lymphocytes, and T cells from secondary lymphoid organs also displayed a decrease in CD69, CD25, PD-1, and Tim-3 activation markers [76]. Accordingly, Th1 and Th17 cell numbers in the draining lymph nodes were lower in PV1-treated mice when compared with their untreated counterparts. Of note, regulatory T cells (T_reg_) were also decreased in the secondary lymphoid organs after PV1 administration [76], suggesting that the overall reduction in T cell activation and cytokine production is not T_reg_-dependent, and does not result from anergy. In fact, antigen-specific restimulation of draining lymph node cells in the presence of PV1 resulted in decreased IFN-γ production, indicating that CD28 blockade by PV1 impairs TH1 lymphocyte ability to secrete cytokines upon antigen reencounter [76].

Altogether, CD28 blockade in AIU is a promising immunotherapeutic strategy, as it seems to act directly in the effector T_H_ subsets responsible for the pathogenesis of the autoimmune uveitis. It is important, however, to better understand the mechanisms of action of CD28 blockade, to ensure specificity and avoid undesired interactions with other co-stimulation pathways.

## 6. Lupus Nephritis

Lupus is a prototypic autoimmune disease resulting from a loss of tolerance to self-antigens. Targeting costimulatory pathways is an emerging therapeutic strategy in lupus [78], particularly lupus nephritis, one of the most frequent and serious manifestations of the disease. In animal models, a CTLA4-Ig fusion protein blocked autoantibody production, prevented lupus nephritis, and prolonged life in NZB/NZW lupus-prone mice [79]. In clinical trials, however, humanized CTLA-4-Ig (abatacept, belatacept) had a limited impact on mild or severe (nephritis) forms of the disease [80,81]. This lack of clear clinical efficacy may be due to the simultaneous blockade by these drugs of CD28 and CTLA-4 pathways, which negatively impacts on the expansion and function of Tregs. Selective blockade of CD28 to preserve the generation of Tregs may therefore be a more efficacious approach for targeting costimulatory pathways in lupus. In NZB/NZW lupus prone mice, 3-month treatment with a pegylated Fab’ antibody fragment against mouse CD28 prevented the development of lupus nephritis in terms of proteinuria and of renal glomerular and interstitial inflammatory changes. Treatment also prolonged overall survival [82], and its effects were sustained for up to 12 weeks after treatment discontinuation. Similarly, anti-CD28 blockade decreased, but not abrogated, the production of anti-double standard DNA antibodies, along with a trend involving a decrease in the number of intra-renal CD138^+^ cells (presumably plasmocytes). The protective effect of the anti-CD28 Fab’ fragment was associated with a decrease in serum levels of soluble CD40L and of intra-renal expression of TNF-alpha and IL6 (reflecting a decrease in renal inflammation), and of CD244. Treatment was also associated with a decrease in the intra-renal expression of FoxP3^+^, with the number of FoxP3^+^ cells being unexpectedly increased in the setting of murine lupus nephritis [83]. In contrast, in mice where CD28 was blocked, the intrarenal expression of the two co-inhibitory molecules PD1 and PDL-1, of GATA3, CD8, and IDO, were increased compared to control mice. The increase was most striking for the immunoregulatory molecule IDO (mainly in renal tubules), which may result from a decrease in intra-renal inflammation. These encouraging data generated in animal models indicate that selective CD28 blockade warrants further assessment in clinical lupus. 

## 7. Conclusions

This review briefly summarizes the data published principally for primate models of major autoimmune diseases affecting young human adults. Controlling in vitro alloreactivity and responses to allografts via selective CD28 targeting was initially assessed in rodent models [16,84], and was subsequently confirmed in experimental allo-transplantation in primates [11,27,85]. The data reviewed above in the field of autoimmunity show a strong consensus for anti-CD28 blockade, which is similar to transplantation models, with a disturbed generation of effector T cells and expansion of regulatory mechanisms (Treg cells, IDO) [11,82]. Altogether, the initial allotransplantation data and the most recent studies in autoimmune models demonstrate an advantage for selectively targeting CD28 over other molecules, as it uniformly blocks both the activation arm and modulates the CD80/86 co-stimulation branches (extended to non-CD28 ligands such as PDL1 [10]), and provides a compelling case for clinical development. 

The maturation of a target molecule from initial concept to a therapeutically accepted entity is typically a long process, and is restricted by known and unpredictable hazards. In this respect, the translation of the selective CD28 antagonists, with encouraging effectiveness in a variety of stringent experimental models of human diseases, was deliberately developed with major safety considerations in mind [22]. The initial option of developing a monovalent Fc-free molecule targeting an antagonist CD28 epitope, being unable to crosslink CD28, and showing no adverse effects in NHP and humans [28,86], has rejuvenated the interest of blocking this major co-stimulation pathway of the immune response. It is also conceivable that selectively targeting T cells instead of multiple cell lineages including non-immune cells, as for anti-CD40, will further enhance the therapeutic safety profile. However, a possible drawback of a strong control of T cell activation is an increased probability to fail in mounting protective immune responses, mainly against viruses. This point has been carefully followed up in the first clinical evaluation [28]. In addition, as for other humanized therapeutic monoclonal antibodies there is a risk that anti-drug antibodies develop in some patients, which if quantitatively important might impact tolerance or activity. Only forthcoming clinical trials can definitively assess the risk-benefit ratio of the various approaches of costimulation blockade, and may ultimately allow fine-tuning to achieve the optimal clinical indications.
